# nce-miR-12220 is a vital regulator for microsporidian to infect honeybee via positive modulation of *ATP-A* and *γ-tubulin* genes

**DOI:** 10.1371/journal.ppat.1014521

**Published:** 2026-08-17

**Authors:** Rui Guo, He Zang, Wenhua Xu, Kaiyao Zhang, Xue Yang, Nian Fan, Yaping Ye, Jianfeng Qiu, Dafu Chen

**Affiliations:** 1 College of Bee Science, Fujian Agriculture and Forestry University, Fuzhou, China; 2 National & Local United Engineering Laboratory of Natural Biotoxin, Fuzhou, China; 3 Apitherapy Research Institute, Fujian Agriculture and Forestry University, Fuzhou, China; Institut Pasteur, FRANCE

## Abstract

Microsporidia rely extensively on host resources, yet how parasite-derived microRNAs coordinate infection remains poorly understood. Here, we investigated the function of nce-miR-12220, a miRNA identified in *Nosema ceranae* spores, during infection of *Apis mellifera* workers. Target prediction, dual-luciferase assays, and fluorescence in situ hybridization were combined with RNA interference and miRNA gain- and loss-of-function experiments. nce-miR-12220 interacted sequence-specifically with binding regions in *ATP-A* and *γ-tubulin* and was detected in infected honeybee midgut epithelial cells. Silencing either target gene reduced expression of the *N. ceranae* virulence-associated gene *NcRBL* and improved worker survival relative to the scramble control. In infected workers, nce-miR-12220 overexpression increased endogenous *ATP-A* and *γ-tubulin* transcript abundance, whereas inhibition produced the opposite effect. Overexpression also reduced expression of the Toll pathway-associated genes *Cactus* and *dorsal* and the antimicrobial peptide genes *Defensin* and *Hymenoptaecin*, increased *N. ceranae* spore load and sucrose consumption, and decreased midgut ATP content. Inhibition of nce-miR-12220 reversed these responses and reduced parasite burden. Survival after nce-miR-12220 manipulation changed in the predicted direction but did not reach statistical significance. Together, these findings identify nce-miR-12220 as a parasite-derived regulator that promotes *N. ceranae* proliferation while reshaping host immune and energetic responses, and suggest that this microRNA and its target network may provide candidates for controlling bee nosemosis.

## Author summary

Honey bees are essential pollinators, but their health is threatened by *Nosema ceranae*, a microscopic parasite that multiplies inside cells of the bee gut. This parasite has lost many metabolic abilities and must exploit its host to obtain energy and complete its life cycle. We investigated whether a small parasite-produced RNA molecule, nce-miR-12220, helps coordinate this process. We found the molecule inside infected honeybee gut cells and showed that it interacts with two genes involved in energy use and cell structure, *ATP-A* and *γ-tubulin*. Increasing nce-miR-12220 promoted parasite spore production, weakened the expression of several bee immune-defense genes, increased sugar consumption, and reduced energy stored as ATP in the gut. Blocking nce-miR-12220 produced the opposite effects and lowered the parasite burden. Silencing either target gene also reduced expression of a parasite virulence-associated gene and improved bee survival. However, altering nce-miR-12220 alone did not produce a statistically significant survival difference during the experiment. Our findings suggest that *N. ceranae* uses this small RNA to coordinate parasite growth with changes in host immunity and energy use. This regulatory network may offer new targets for developing non-antibiotic approaches to control bee nosemosis.

## Introduction

*Apis mellifera*, one of the most important pollinators worldwide, plays an indispensable role in maintaining the functionality of natural ecosystems and ensuring agricultural productivity. As a primary pollinator, its services are essential for approximately 75% of crops, underpinning an estimated global economic value ranging from $235 billion to $577 billion [1], while also promoting the reproduction of flowering plants that sustain approximately 90% of wild plant diversity [2,3]. However, *A. mellifera* is subjected to various biotic and abiotic stresses, including parasites, pathogens, and pesticides, leading to considerable declines in bee populations [4,5]. These challenges not only threaten the survival of bees but also pose significant risks to global food security and biodiversity. Among these factors, *Nosema ceranae* is recognized as a major contributor to bee population losses [6]. Consequently, protecting and managing the diversity and health of *A. mellifera* populations have become critical priorities [7].

*N. ceranae* is a specialized intracellular parasitic microsporidian belonging to the fungal kingdom, primarily infecting the midgut epithelial cells of bees [8]. It germinates in the host gut lumen by producing resistant spores and utilizes a polar filament to inject infectious sporoplasm into host cells [6,9]. Initially discovered in *Apis cerana*, *N. ceranae* has since spread to *A. mellifera* and other bee species worldwide due to global beekeeping practices [6,10]. The infection process involves spore ingestion, polar filament ejection, host cell invasion, and intracellular proliferation, giving rise to dysregulation of gut function, impaired nutritional absorption, immune suppression, and a significant reduction in bee lifespan [9,11]. Current control measures against this fungal parasite rely on limited antibiotics, such as fumagillin. However, the risk of resistance and ecological toxicity underscores the urgency of developing novel intervention strategies, including RNA interference and natural product interventions [12–14].

In the molecular interplay between hosts and pathogens, microRNAs (miRNAs), a class of non-coding RNAs approximately 20–24 nucleotides in length, play crucial roles by targeting the 3’ UTR regions of mRNAs through base pairing and mediating post-transcriptional gene silencing. They are essential molecules in regulating host immune response and pathogen infection. For instance, in interactions between bees and microsporidians, the host miR-989 targets mRNAs of immune-related genes, such as those involved in antibacterial peptide synthesis pathways, inhibiting their translation and thereby diminishing host immune defenses, which enhances the proliferation of *N. ceranae* [15]. Conversely, pathogen-secreted miR-21-x targets host mitochondrial oxidative phosphorylation pathway-related genes, potentially undermining the host cell’s anti-infection capabilities by disrupting energy metabolism [16]. This bidirectional regulatory mechanism underscores the strategic role of miRNAs in cross-kingdom regulation.

Recent advances in the study of *N. ceranae* miRNAs have identified a series of miRNAs (e.g., miR-598-y, miR-252-y, miR-92-x, and miR-3654-y) whose expression levels exhibit dynamic changes throughout the infection cycle [17,18]. Experimental evidence confirms that these miRNAs not only participate in regulating genes relative to fungal proliferation, such as those encoding critical spore-forming proteins [18], but also enter the host cytoplasm via exosomal pathways, targeting genes associated with ion binding, transmembrane transport, and immune signal transduction [19,20]. For example, knockdown of *N. ceranae Dicer* and *Argonaute* genes significantly inhibits miRNA production, leading to a reduction of over 50% in parasite reproduction, suggestive of a vital role for fungal miRNAs during the infection process [19,21]. Additionally, investigation of miRNA-mRNA interactions between host and microsporidian has revealed that host miRNAs, such as miR-676-y, can inversely target virulence factor genes in *N. ceranae*, forming a complex defense-counter-defense molecular network [22,23].

The regulatory mechanisms of miRNAs in the *N. ceranae* infection of honeybees remains unclear until present. In our previous studies, our team identified ten miRNAs, including nce-miR-12220, in the *N. ceranae* spores on basis of small RNA-seq and bioinformatics [23], followed by molecular validation, expression profile analysis, and target annotation [24]. In this current work, the direct binding of nce-miR-12220 to its key target genes was verified. Additionally, overexpression and knockdown of nce-miR-12220 were conducted to dissect its functions, followed by RNAi of key targets to explore the underlying mechanism. Our finding will not only illustrate the miRNA-mediated regulatory mechanism underlying the N. ceranae infection of honeybees at molecular level, but also provide novel candidates and strategy for diagnosis and control of bee nosemosis.

## Materials and methods

### Honey bee colonies, parasite source, and common rearing conditions

Newly emerged *A. mellifera* workers were obtained from three independent colonies maintained at the experimental apiary of Fujian Agriculture and Forestry University, Fuzhou, China. Workers originating from different colonies were kept in separate cages throughout the experiments. Unless otherwise stated, each treatment comprised three independent cages, with one cage established from each source colony. The cage/colony combination was therefore considered one biological replicate. Functional experiments used 35 workers per cage, whereas the initial infection time-course experiment used 50 workers per cage. Bees were maintained at 34 ± 0.5°C and 75% relative humidity and were provided with sterile 50% (w/v) sucrose solution.

*N. ceranae* spores were obtained from a laboratory stock established in previous work and deposited at the China General Microbiological Culture Collection Center (CGMCC accession no. 28110). Midguts were dissected from donor workers, homogenized in sterile water, and examined by light microscopy at 400 × magnification. Only samples containing abundant *N. ceranae* spores were selected for subsequent spore purification.

### Purification of *N. ceranae* spores and oral inoculation

Midguts containing abundant spores were pooled in RNase-free microcentrifuge tubes containing 500 μL sterile water and homogenized using a high-throughput tissue grinder (Meibi, China). Homogenates were centrifuged at 13,000 × g for 10 min at 4°C. The resulting pellets were purified using a discontinuous Percoll gradient composed of 25%, 50%, 75%, and 100% Percoll. Following centrifugation at 15,000 × g for 40 min at 4°C, the spore-containing material was recovered, and the gradient-purification step was repeated once. Purified spores were washed in 500 μL sterile water by centrifugation at 5,000 × g for 5 min at 4°C, resuspended in 500 μL sterile water, and stored at 4°C. Spore concentration was determined using a hemocytometer.

For infection, capped brood combs from the three source colonies were transferred to the controlled incubator described above. Newly emerged workers were collected separately from each colony and fasted for 2 h. Each worker assigned to an infected group was then fed individually with 5 μL of 50% (w/v) sucrose solution containing 1 × 10^6^ *N. ceranae* spores. Workers in the uninfected control group received an equal volume of spore-free 50% sucrose solution. Two hours after inoculation, each cage was supplied with 5 mL sterile 50% sucrose solution, which was replaced every 24 h.

### Infection time-course experiment

To define the temporal expression pattern of *ATP-A* during infection, workers were assigned to an uninfected control group or an *N. ceranae*-infected group. Each treatment included three colony-derived cages containing 50 workers per cage. Midguts were collected daily from 1 to 12 dpi. At each time point, three midguts from one cage were pooled in a single RNase-free 1.5-mL microcentrifuge tube and constituted one biological sample. Samples were immediately frozen in liquid nitrogen and stored at −80°C until RNA extraction.

### Target-gene RNAi experiment

To determine the roles of *ATP-A* and *γ-tubulin* during infection, gene-specific siRNAs targeting *ATP-A* (GenBank accession XM_024475731.1) or *γ-tubulin* (GenBank accession XM_024474433.1), together with a non-targeting siRNA control, were synthesized by Genechem Biotech Co., Ltd. (Shanghai, China). These siRNAs were designated si-ATP-A, si-γ-tubulin, and si-scramble, respectively. After the 2-h fasting period, each worker received 5 μL of 50% sucrose solution containing 1 × 10^6^ *N. ceranae* spores and 1.5 μg of si-ATP-A, si-γ-tubulin, or si-scramble. These groups were designated *N. ceranae* + si-ATP-A, *N. ceranae* + si-γ-tubulin, and *N. ceranae* + si-scramble, respectively. For molecular analyses, the expression levels of *ATP-A*, *γ-tubulin*, and *NcRBL* (GenBank accession NW_020169305.1) were measured from 1 to 6 dpi in the three RNAi groups. At each time point, three midguts from each cage were pooled as one biological replicate. For the physiological analyses, an uninfected group and an *N. ceranae*-only group were included in addition to the three RNAi groups. Sucrose consumption and worker survival were monitored from 1 to 12 dpi. Each treatment comprised three independent cages containing 35 workers per cage.

### nce-miR-12220 gain- and loss-of-function experiment

The raw miRNA-seq data were deposited in the NCBI Sequence Read Archive (SRA) under BioProject accession PRJNA395137. nce-miR-12220 was one of the ten miRNAs identified in this dataset and was mapped to scaffold NW_003312212 at positions 610–859. Its mature sequence was 5′-UUUAAUUGUGAAACAUGUCUUUAGG-3′. A synthetic nce-miR-12220 mimic (M-miR-12220), an antisense inhibitor (I-miR-12220), and the corresponding negative controls (M-NC and I-NC) were synthesized by Shanghai Jima Pharmaceutical Technology Co., Ltd. (Shanghai, China). Overexpression and inhibition were performed using the previously established feeding procedures described in references [25,26]. Infected workers were assigned to four groups: *N. ceranae* + M-miR-12220, *N. ceranae* + M-NC, *N. ceranae* + I-miR-12220, and *N. ceranae* + I-NC.

Each group comprised three independent colony-derived cages containing 35 workers per cage. For molecular analyses, the expression levels of nce-miR-12220, *ATP-A*, *γ-tubulin*, *Cactus*, *dorsal*, *Defensin*, and *Hymenoptaecin* were measured from 1 to 6 dpi. At each time point, three midguts from each cage were pooled as one biological replicate. For phenotypic analyses, worker survival, *N. ceranae* spore load, sucrose consumption, and midgut ATP content were evaluated from 1 to 12 dpi.

### RNA extraction, reverse transcription, and RT-qPCR

Total RNA was extracted from pooled midgut samples using a commercial RNA extraction kit. For nce-miR-12220 quantification, reverse transcription was performed using a sequence-specific stem-loop primer. For protein-coding transcripts, first-strand cDNA was synthesized using oligo (dT) and random primers. Quantitative PCR was performed on a QuantStudio 3 Real-Time PCR System (Applied Biosystems, USA) using the reaction system and cycling conditions described previously [27]. *U6* was used as the internal control for nce-miR-12220, whereas *β-actin* was used as the internal control for mRNA targets. Relative expression was calculated using the 2 − ΔΔCt method. Each biological sample was analyzed in three technical qPCR replicates, and the mean Ct value of the technical replicates was used for statistical analysis. Primer sequences and oligonucleotide information were provided in S1 Table.

### Dual-luciferase reporter assay

Approximately 300-bp regions containing the predicted nce-miR-12220-binding sites in *ATP-A* and *γ-tubulin* were amplified and verified by Sanger sequencing. Complementary oligonucleotides carrying either the wild-type target sequence or a mutated binding sequence were synthesized. For annealing, 2 μL aliquots of the forward and reverse oligonucleotides (1 μg/μL) were combined with 46 μL of Oligo Annealing Buffer, heated at 90°C for 3 min, and incubated at 37°C for 15 min. A 1-μL aliquot of the annealed product was ligated overnight into 50 ng of the pmirGLO vector, which had been digested with ScaI and XhoI, using T4 DNA ligase (Promega, China). The ligation products were transformed into competent *Escherichia coli* cells (Qingke Biology, China). Transformants were selected on ampicillin-containing medium and verified by Sanger sequencing using the pCMV5-R primer. The resulting plasmids were designated pmirGLO-ATP-A-miR-12220-wt, pmirGLO-ATP-A-miR-12220-mut, pmirGLO-γ-tubulin-miR-12220-wt, and pmirGLO-γ-tubulin-miR-12220-mut.

HEK-293T cells were cultured in high-glucose Dulbecco’s modified Eagle medium supplemented with fetal bovine serum and antibiotics at 37°C in 5% CO2. Cells were seeded in 48-well plates and transfected at 90–95% confluence using a lipofection reagent (Yisheng, China). For *ATP-A*, the four transfection conditions were mimic-miR-12220 + wild-type reporter, M-NC + wild-type reporter, mimic-miR-12220 + mutant reporter, and M-NC + mutant reporter. The same four combinations were established for *γ-tubulin*. At 24 h after transfection, cells were lysed on ice for 5 min in 100 μL of lysis buffer, and the lysates were centrifuged at 10,000 × g for 1 min. Firefly and Renilla luciferase activities were measured sequentially using a dual-luciferase reporter assay kit (Yisheng, China) and a chemiluminescence detector (Promega, China). Relative reporter activity was calculated as the firefly-to-Renilla luciferase ratio. Three independent transfection experiments were performed.

### Fluorescence *in situ* hybridization of nce-miR-12220

At 6 dpi, midguts were dissected from *N. ceranae*-infected and uninfected *A. mellifera* workers (*n* = 3) and submitted to Wuhan Servicebio Technology Co., Ltd. (Wuhan, China) for paraffin embedding, sectioning, and slide preparation. An antisense oligonucleotide probe complementary to nce-miR-12220 (5′-CCTAAAGACATGTTTCACAATTAAA-3′) was synthesized by Fuzhou Shangya Biotechnology Co., Ltd. (Fuzhou, China), purified by high-performance liquid chromatography, and labeled with Cy3 at the 5′ end. RNA fluorescence in situ hybridization was performed using the Fluorescence in Situ Hybridization Kit for RNA (Beyotime Biotechnology, Shanghai, China) according to the manufacturer’s instructions. Briefly, paraffin sections were deparaffinized, rehydrated through a graded ethanol series, permeabilized with proteinase K, post-fixed with 4% paraformaldehyde, treated with hydrochloric acid, and acetylated to reduce nonspecific background. After prehybridization, the sections were incubated with the Cy3-labeled probe in hybridization solution under RNase-free and light-protected conditions. Following stringent washing with the supplied wash buffers, nuclei were counterstained with DAPI. Sections were mounted with antifade mounting medium and examined by fluorescence microscopy. The Cy3 signal was used to indicate nce-miR-12220 localization, and infected and uninfected sections were imaged using identical acquisition settings.

### Spore load quantification

At each time point, one worker was sampled from each cage. The dissected midgut was homogenized in 200 μL of sterile water using a high-throughput tissue grinder, after which 800 μL of sterile water was added. A 10 μL aliquot of the suspension was loaded into a hemocytometer, and spores were counted by light microscopy at 400 × magnification. One midgut from each of the three independent cages was analyzed per treatment and time point. Spore counts were log2-transformed before statistical analysis.

### Sucrose solution consumption

Sucrose consumption was measured at the cage level. A feeder containing 5 mL sterile 50% (w/v) sucrose solution was weighed at the start of each 24-h interval and reweighed immediately before replacement. Daily consumption was corrected for the number of workers alive in the corresponding cage and expressed as milligrams of 50% (w/v) sucrose solution consumed per worker per day. The cage was the experimental unit, with three cages analyzed per treatment.

### Survival analysis

Survival cohorts contained 35 workers per cage and three cages per treatment, corresponding to 105 workers per treatment at the start of follow-up. The number of living workers was recorded every 24 h from 1 to 12 dpi immediately before sucrose-solution replacement, and dead workers were removed after each observation. Survival was expressed as the proportion of workers remaining alive at each observation time and displayed as Kaplan–Meier survival curves.

### Measurement of ATP content in worker midguts

Midgut ATP content was quantified using the Enhanced ATP Assay Kit (Beyotime Biotechnology, Shanghai, China) according to the manufacturer’s instructions. Sample preparation was performed on ice. Midgut tissue was homogenized in ATP detection lysis buffer at a ratio of 100–200 μL lysis buffer per 20 mg tissue. Homogenates were centrifuged at 12,000 × g for 5 min at 4°C, and the supernatants were collected for ATP measurement. ATP standards were prepared in ATP detection lysis buffer over a concentration range of 0.01–10 μM. The ATP detection reagent was diluted 1:4 with the supplied diluent. For each determination, 100 μL of working solution was added to a detection well and equilibrated at room temperature for 3–5 min to reduce background luminescence. A 20 μL aliquot of each sample or ATP standard was then added to the well and mixed immediately, after which luminescence was recorded using a luminometer. ATP concentrations were calculated from the standard curve and expressed as μM. Three independent biological replicates were analyzed for each treatment and time point.

### Statistical analysis

Statistical analyses were performed using GraphPad Prism 9 (GraphPad Software, San Diego, CA, USA). Unless otherwise stated, data were presented as the mean ± standard deviation (SD) of three biological replicates. *ATP-A* infection time-course data were analyzed by one-way analysis of variance followed by Tukey’s multiple-comparisons test. Dual-luciferase reporter data were analyzed using two-tailed, unpaired Student’s *t*-tests. Longitudinal gene-expression, sucrose-consumption, spore-load, and midgut ATP data were analyzed using mixed-effects models fitted by restricted maximum likelihood. Treatment, dpi, and the treatment × dpi interaction were included as fixed effects, and repeated observations from the same cage/colony replicate were accounted for in the model. Šídák’s multiple-comparisons test was used for comparisons between treatment groups at corresponding time points. In the figures, bracketed *P* values were used to represent the overall difference between the indicated groups across the examined time course, whereas asterisks above individual time points were used to indicate the corresponding Šídák-adjusted pairwise comparisons. Survival distributions were estimated using the Kaplan–Meier method and compared using the log-rank (Mantel–Cox) test. All tests were two-sided, and *P* < 0.05 was considered statistically significant.

## Results

### nce-miR-12220 interacted with target genes *ATP-A* and *γ-tubulin*

**Fig 1:**
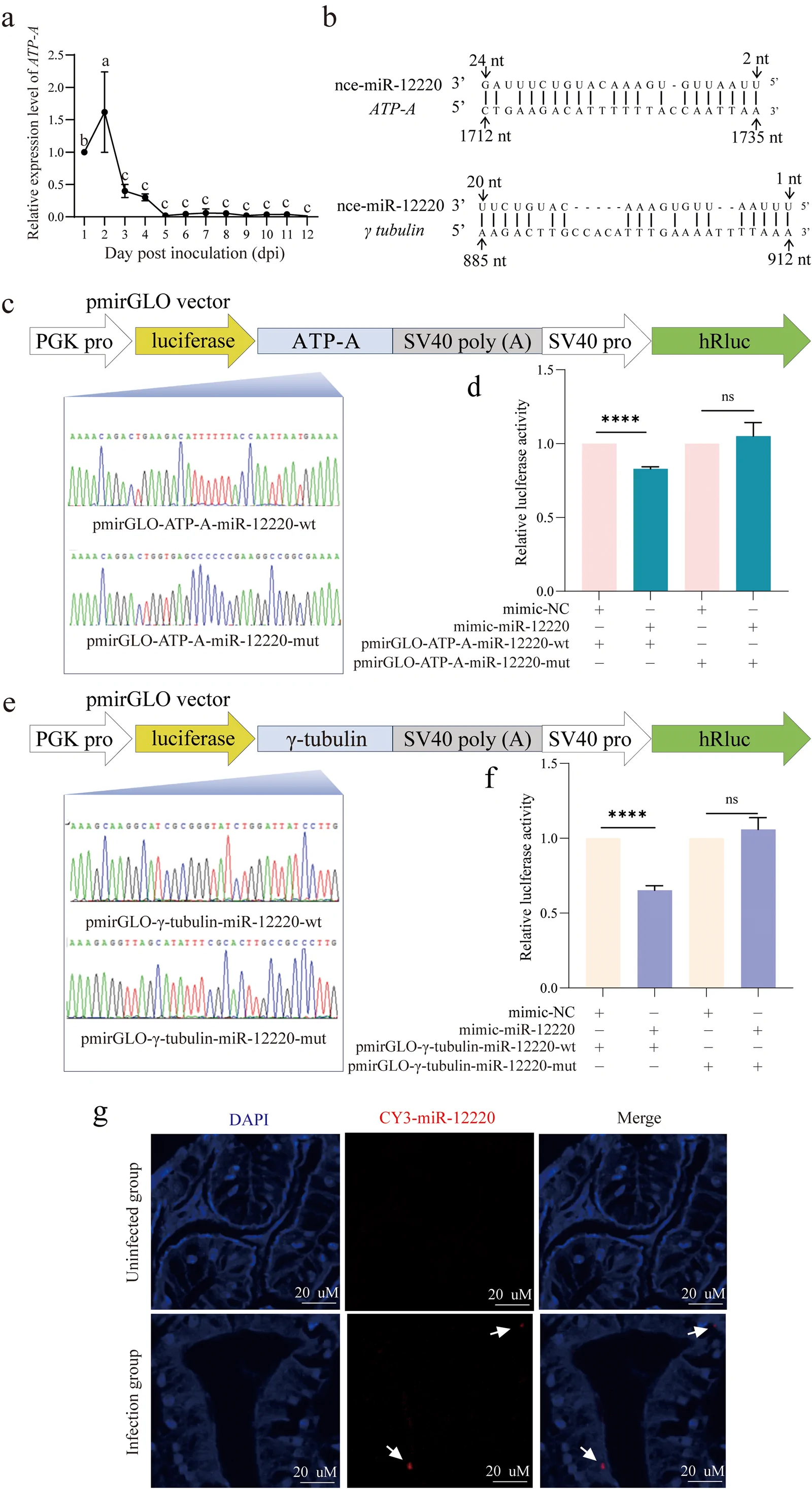
*ATP-A* expression pattern, validation of nce-miR-12220 interactions with *ATP-A* and *γ-tubulin*, and localization of nce-miR-12220 in the honeybee midgut during *N. ceranae* infection. **(a)** Relative expression levels of *ATP-A* in *A. mellifera* workers during *N. ceranae* infection from 1 to 12 days post-infection (dpi). Different letters indicate significant differences among time points, as determined by one-way ANOVA followed by Tukey’s multiple-comparison test. **(b)** Predicted base-pairing between nce-miR-12220 and the target sequences in *ATP-A* and *γ-tubulin*. **(c)** Schematic representation of the pmirGLO-ATP-A reporter constructs and Sanger sequencing confirmation of the wild-type and mutant *ATP-A* binding regions. **(d)** Dual-luciferase reporter assay validating the interaction between nce-miR-12220 and the predicted binding site in *ATP-A*. **(e)** Schematic representation of the pmirGLO-*γ-tubulin* reporter constructs and Sanger sequencing confirmation of the wild-type and mutant *γ-tubulin* binding regions. **(f)** Dual-luciferase reporter assay validating the interaction between nce-miR-12220 and the predicted binding site in *γ-tubulin*. **(g)** Representative RNA fluorescence *in situ* hybridization images of midgut sections from uninfected and *N. ceranae*-infected *A. mellifera* workers at 6 dpi. For panels (d) and **(f)**, statistical significance was assessed using Student’s *t*-test. Data are presented as the mean ± SD of three biological replicates. ns, *P* > 0.05; ****, *P* < 0.0001.

The expression pattern of *ATP-A* was examined in *A. mellifera* workers during *N. ceranae* infection. *ATP-A* expression increased at 2 dpi, reaching its highest level among the examined time points, and then declined markedly from 3 to 12 dpi. Its expression remained relatively low from 6 to 12 dpi (Fig 1a). Therefore, we considered 1–6 dpi to be the critical regulatory window in which nce-miR-12220 is most likely to exert its direct effects on target genes and host immune responses.

Target prediction identified complementary binding sites for nce-miR-12220 within *ATP-A* and *γ-tubulin*. The predicted binding site was located at nucleotides 1712–1735 in *ATP-A* and 885–912 in *γ-tubulin* (Fig 1b). To validate these predicted interactions, wild-type and mutant reporter constructs containing the corresponding binding regions were generated. Sanger sequencing confirmed the correct construction of recombinant plasmids carrying either the wild-type or mutated binding regions of *ATP-A* and *γ-tubulin* (Fig 1c, 1e). Dual-luciferase reporter assays further validated these interactions. Co-transfection with mimic-miR-12220 significantly reduced the relative luciferase activity of the wild-type *ATP-A* reporter compared with the M-NC group, while mutation of the predicted binding site abolished this effect (Fig 1d). A similar result was obtained for *γ-tubulin*: mimic-miR-12220 significantly decreased the luciferase activity of the wild-type reporter, whereas no significant difference was detected for the mutant reporter (Fig 1f). These findings demonstrate that nce-miR-12220 interacts with *ATP-A* and *γ-tubulin* through the predicted binding sites in a sequence-dependent manner.

RNA fluorescence in situ hybridization was then used to determine whether nce-miR-12220 could be detected within host midgut cells during *N. ceranae* infection. At 6 dpi, Cy3-positive signals corresponding to nce-miR-12220 were observed within the midgut epithelial cells of infected *A. mellifera* workers, whereas no obvious signal was detected in the uninfected control group (Fig 1g). These findings provide spatial evidence that the *N. ceranae*-derived nce-miR-12220 is present within honeybee midgut cells during infection.

### Target gene silencing significantly affected the expression of *N. ceranae RBL* gene

**Fig 2:**
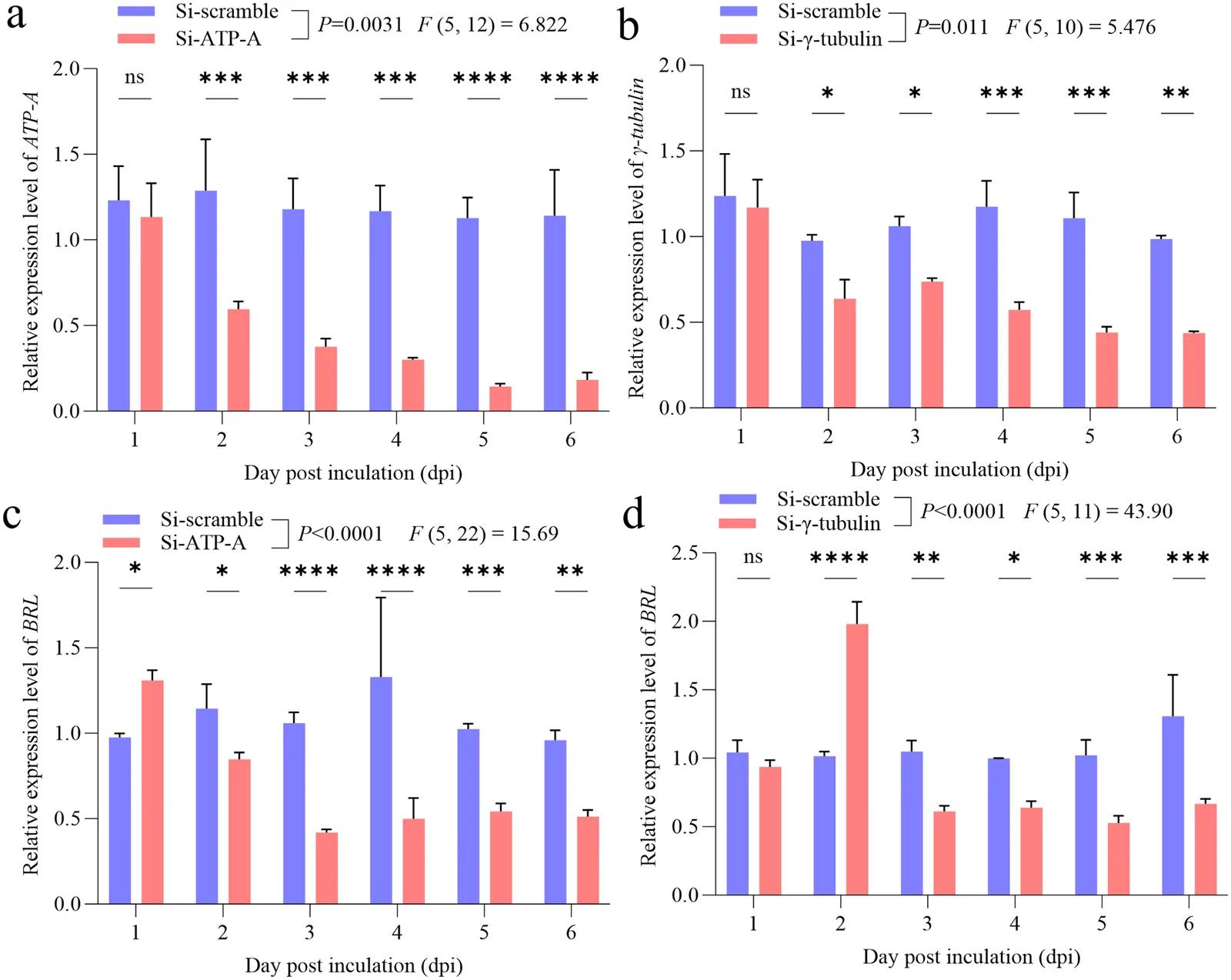
Effects of *ATP-A* and *γ-tubulin* silencing on *NcRBL* expression in *A. mellifera* workers during *N. ceranae* infection. **(a)** Relative expression level of *ATP-A* following RNAi-mediated *ATP-A* silencing from 1 to 6 days post-infection (dpi). **(b)** Relative expression level of *γ-tubulin* following RNAi-mediated *γ-tubulin* silencing from 1 to 6 dpi. **(c)** Relative expression level of *NcRBL* after *ATP-A* silencing. **(d)** Relative expression level of *NcRBL* after *γ-tubulin* silencing. Statistical significance was assessed using a mixed-effects model for repeated-measures data, followed by Šídák’s multiple-comparisons test. Data are presented as the mean ± SD of three biological replicates. ns, *P* > 0.05; *, *P* < 0.05; **, *P* < 0.01; ***, *P* < 0.001; ****, *P* < 0.0001.

To assess the functional roles of the predicted target genes during *N. ceranae* infection, *ATP-A* and *γ-tubulin* were individually silenced in infected *A. mellifera* workers. *ATP-A* expression was significantly reduced in the si-*ATP-A* group compared with the si-scramble group across the examined period, confirming effective RNAi-mediated knockdown (*P* = 0.0031; Fig 2a). Likewise, *γ-tubulin* expression was significantly lower in the si-*γ-tubulin* group than in the si-scramble group (*P* = 0.011; Fig 2b).

The effect of target-gene silencing on *NcRBL* expression was then examined. Silencing *ATP-A* significantly reduced *NcRBL* expression throughout the infection period relative to the si-scramble group (*P* < 0.0001; Fig 2c). A similar reduction was observed after *γ-tubulin* silencing, with *NcRBL* expression remaining significantly lower than that in the si-scramble group across the examined period (*P* < 0.0001; Fig 2d). These results suggest that *ATP-A* and *γ-tubulin* positively contribute to *NcRBL* expression during *N. ceranae* infection.

### Target gene interference influenced host survival

**Fig 3:**
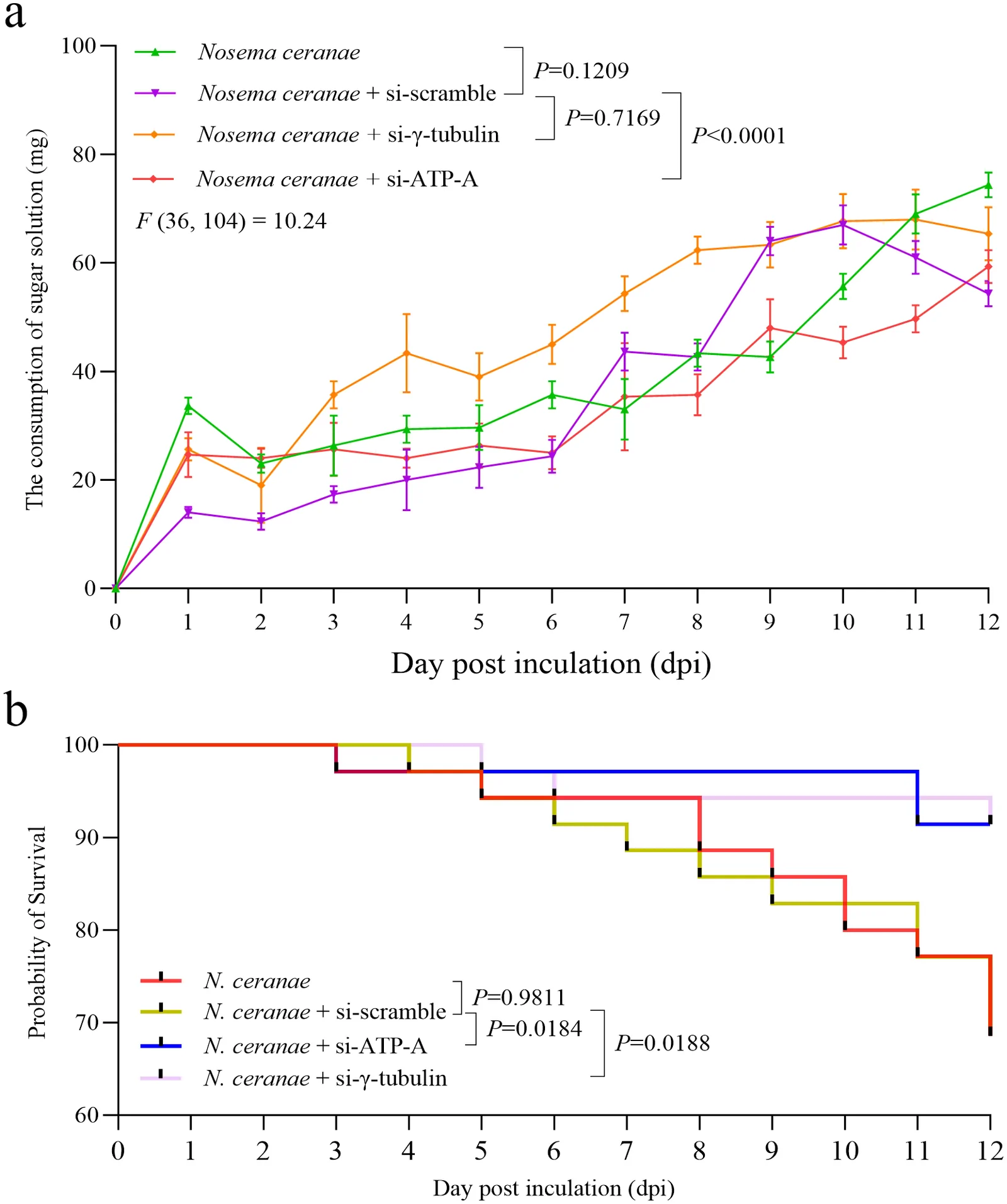
Effects of *ATP-A* and *γ-tubulin* silencing on sucrose consumption and survival of *A. mellifera* workers during *N. ceranae* infection. **(a)** Consumption of 50% (w/v) sucrose solution, expressed as milligrams per worker per day, by infected workers from 1 to 12 dpi following *ATP-A* or *γ-tubulin* silencing. Statistical significance was assessed using a mixed-effects model for repeated-measures data, followed by Dunnett’s multiple comparisons test. Data are presented as the mean ± SD of three biological replicates. **(b)** Kaplan–Meier survival curves of infected workers following *ATP-A* or *γ-tubulin* silencing. Survival distributions were compared using the log-rank (Mantel–Cox) test.

To further assess the effects of target-gene silencing during *N. ceranae* infection, sucrose consumption and survival of infected *A. mellifera* workers were monitored from 1 to 12 dpi. Sucrose consumption in the *N. ceranae*-infected group generally increased over time. No significant overall difference was detected between the *N. ceranae* group and the *N. ceranae* + siRNA-scramble group (*P* = 0.1209; Fig 3a), indicating that the scramble siRNA treatment did not significantly affect sucrose intake. Likewise, *γ-tubulin* silencing did not significantly alter overall sucrose consumption compared with the siRNA-scramble control (*P* = 0.7169; Fig 3a). In contrast, *ATP-A* silencing significantly altered sucrose consumption across the examined period (*P* < 0.0001; Fig 3a).

Survival analysis showed no significant difference between the *N. ceranae* group and the *N. ceranae* + si-scramble group (*P* = 0.9811; Fig 3b). Compared with the si-scramble control, *ATP-A* silencing significantly improved worker survival during *N. ceranae* infection (*P* = 0.0184; Fig 3b). Similarly, *γ-tubulin* silencing significantly increased survival probability relative to the si-scramble group (*P* = 0.0188; Fig 3b). These results demonstrate that *ATP-A* and *γ-tubulin* contribute to host physiological responses during *N. ceranae* infection, with silencing of either gene improving worker survival.

### nce-miR-12220 modulated the expression of *ATP-A* and *γ-tubulin* during *N. ceranae* infection

**Fig 4:**
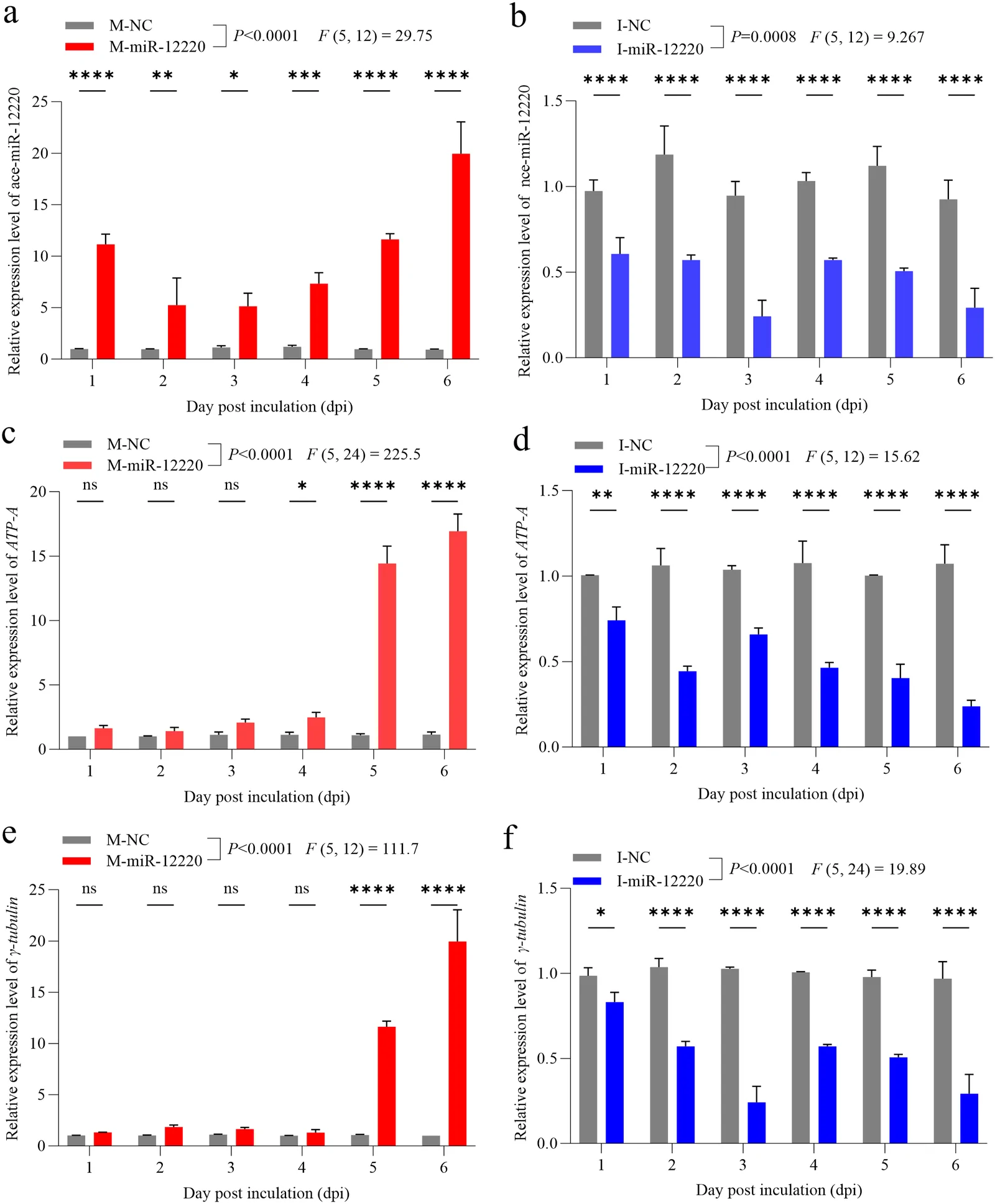
Effects of nce-miR-12220 overexpression and knockdown on *ATP-A* and *γ-tubulin* expression in *A. mellifera* workers during *N. ceranae* infection. **(a-b)** Relative expression level of nce-miR-12220 following M-miR-12220 and I-miR-12220treatment from 1 to 6 dpi. **(c-d)** Relative expression levels of ATP-A following nce-miR-12220 overexpression and knockdown. **(e-f)** Relative expression level of *γ-tubulin* following nce-miR-12220 overexpression and knockdown. Statistical significance was assessed using a mixed-effects model for repeated-measures data, followed by Šídák’s multiple-comparisons test. Data are presented as the mean ± SD of three biological replicates. ns, *P* > 0.05; *, *P* < 0.05; **, *P* < 0.01; ***, *P* < 0.001; ****, *P* < 0.0001.

To determine whether nce-miR-12220 affects the expression of its predicted target genes during *N. ceranae* infection, nce-miR-12220 was manipulated in infected *A. mellifera* workers using a mimic or inhibitor. Compared with the M-NC group, nce-miR-12220 expression was significantly increased overall in the M-miR-12220 group (*P* < 0.0001), with significant increases detected at all examined time points (Fig 4a). Conversely, nce-miR-12220 expression was significantly reduced overall in the I-miR-12220 group relative to the I-NC group (*P* = 0.0008), and significant decreases were observed from 1 to 6 dpi (Fig 4b). These results effective overexpression and knockdown of nce-miR-12220, respectively.

The effects of nce-miR-12220 manipulation on *ATP-A* and *γ-tubulin* expression were then evaluated. Overall, overexpression of nce-miR-12220 significantly increased *ATP-A* expression compared with the M-NC group (*P* < 0.0001). Pairwise comparisons showed significant increases from 3 to 6 dpi, whereas no significant differences were detected at 1 or 2 dpi (Fig 4c). Inhibition of nce-miR-12220 significantly reduced *ATP-A* expression (*P* < 0.0001), with significant decreases observed at all examined time points (Fig 4d).

A similar overall pattern was observed for *γ-tubulin*. nce-miR-12220 overexpression significantly increased *γ-tubulin* expression (*P* < 0.0001), although significant pairwise increases were detected only at 5 and 6 dpi (Fig 4e). In contrast, inhibition of nce-miR-12220 significantly decreased *γ-tubulin* expression (*P* < 0.0001), with significant reductions observed from 1 to 6 dpi (Fig 4f). These findings indicate that nce-miR-12220 positively influences *ATP-A* and *γ-tubulin* expression during *N. ceranae* infection, with the effects of overexpression becoming more pronounced at later infection stages.

### nce-miR-12220 participated in modulating host response to *N. ceranae* invasion through regulating the Toll-like signaling pathway and antimicrobial peptide gene expression

**Fig 5:**
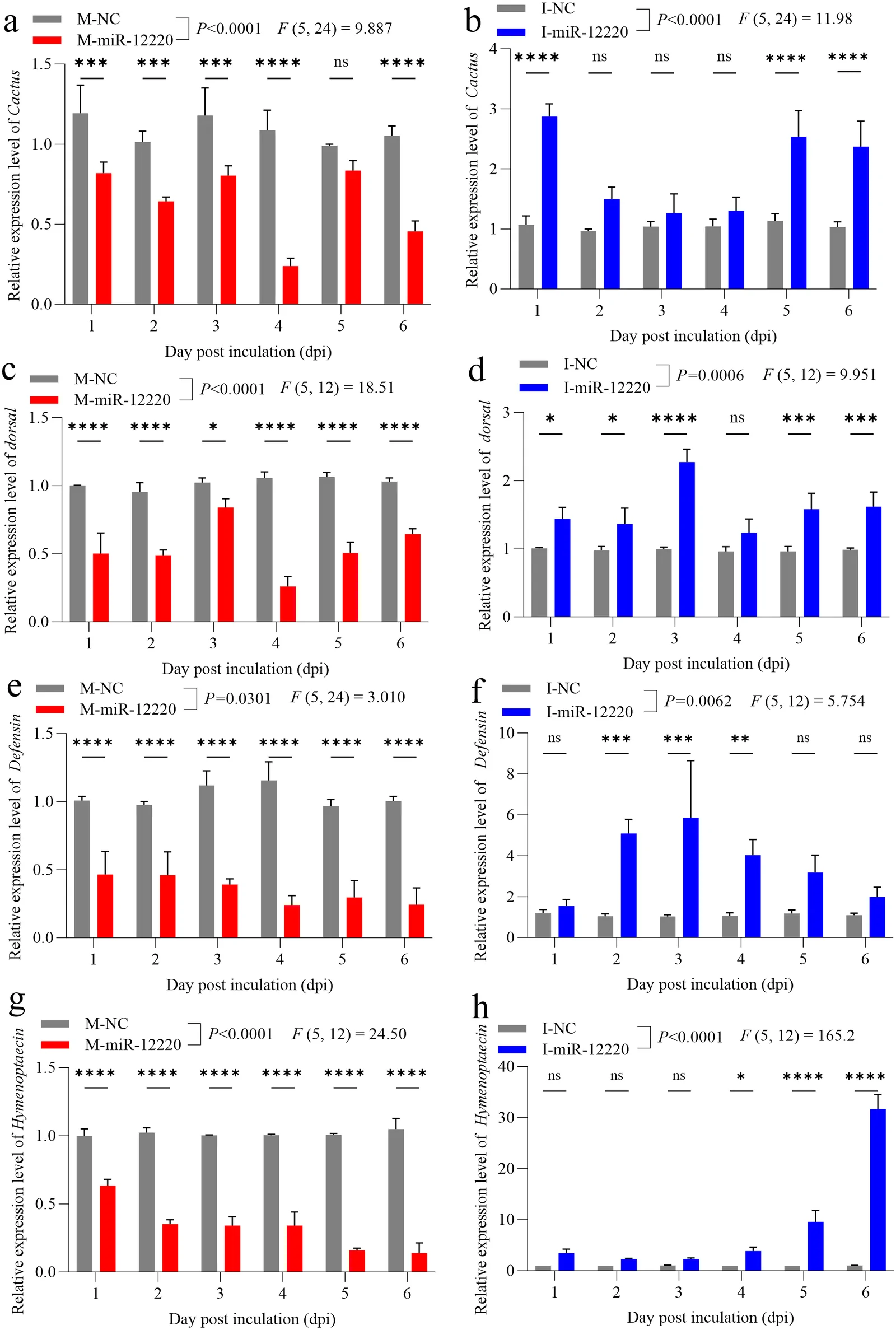
Effects of nce-miR-12220 overexpression and knockdown on immune-related gene expression in *A. mellifera* workers during *N. ceranae* infection. **(a-b)** Relative expression level of Cactus following nce-miR-12220 overexpression and knockdown from 1 to 6 dpi. **(c-d)** Relative expression level of *dorsal* following nce-miR-12220 overexpression and knockdown. **(e-f)** Relative expression level of *Defensin* following nce-miR-12220 overexpression and knockdown. **(g-h)** Relative expression level of *Hymenoptaecin* following nce-miR-12220 overexpression and knockdown. Statistical significance was assessed using a mixed-effects model for repeated-measures data, followed by Šídák’s multiple-comparisons test. Data are presented as the mean ± SD of three biological replicates. ns, *P* > 0.05; *, *P* < 0.05; **, *P* < 0.01; ***, *P* < 0.001; ****, *P* < 0.0001.

To determine whether nce-miR-12220 affects host immune responses during *N. ceranae* infection, the expression levels of genes associated with the Toll signaling pathway and AMPs production were examined after nce-miR-12220 overexpression or knockdown. Compared with the M-NC group, nce-miR-12220 overexpression significantly reduced the overall expression level of *Cactus* across the examined period (*P* < 0.0001; Fig 5a). Conversely, inhibition of nce-miR-12220 significantly increased *Cactus* expression compared with the I-NC group (*P* < 0.0001; Fig 5b).

A similar reciprocal pattern was observed for *dorsal*. Overexpression of nce-miR-12220 significantly decreased *dorsal* expression relative to the M-NC group (*P* < 0.0001; Fig 5c), whereas knockdown of nce-miR-12220 significantly elevated *dorsal* expression compared with the I-NC group (*P* = 0.0006; Fig 5d).

The expression of AMP genes was also altered by nce-miR-12220 manipulation. Compared with the M-NC group, nce-miR-12220 overexpression significantly reduced the overall expression of *Defensin* (*P* = 0.0301; Fig 5e), whereas nce-miR-12220 knockdown significantly increased *Defensin* expression relative to the I-NC group (*P* = 0.0062; Fig 5f). In addition, nce-miR-12220 overexpression significantly decreased *Hymenoptaecin* expression (*P* < 0.0001; Fig 5g), while nce-miR-12220 knockdown markedly increased *Hymenoptaecin* expression (*P* < 0.0001; Fig 5h). These results indicate that nce-miR-12220 manipulation is associated with coordinated changes in the expression of Toll pathway-related genes and AMP genes during *N. ceranae* infection.

### nce-miR-12220 modulated *N. ceranae* spore load, host survival rate and sucrose solution consumption during the infection process

**Fig 6:**
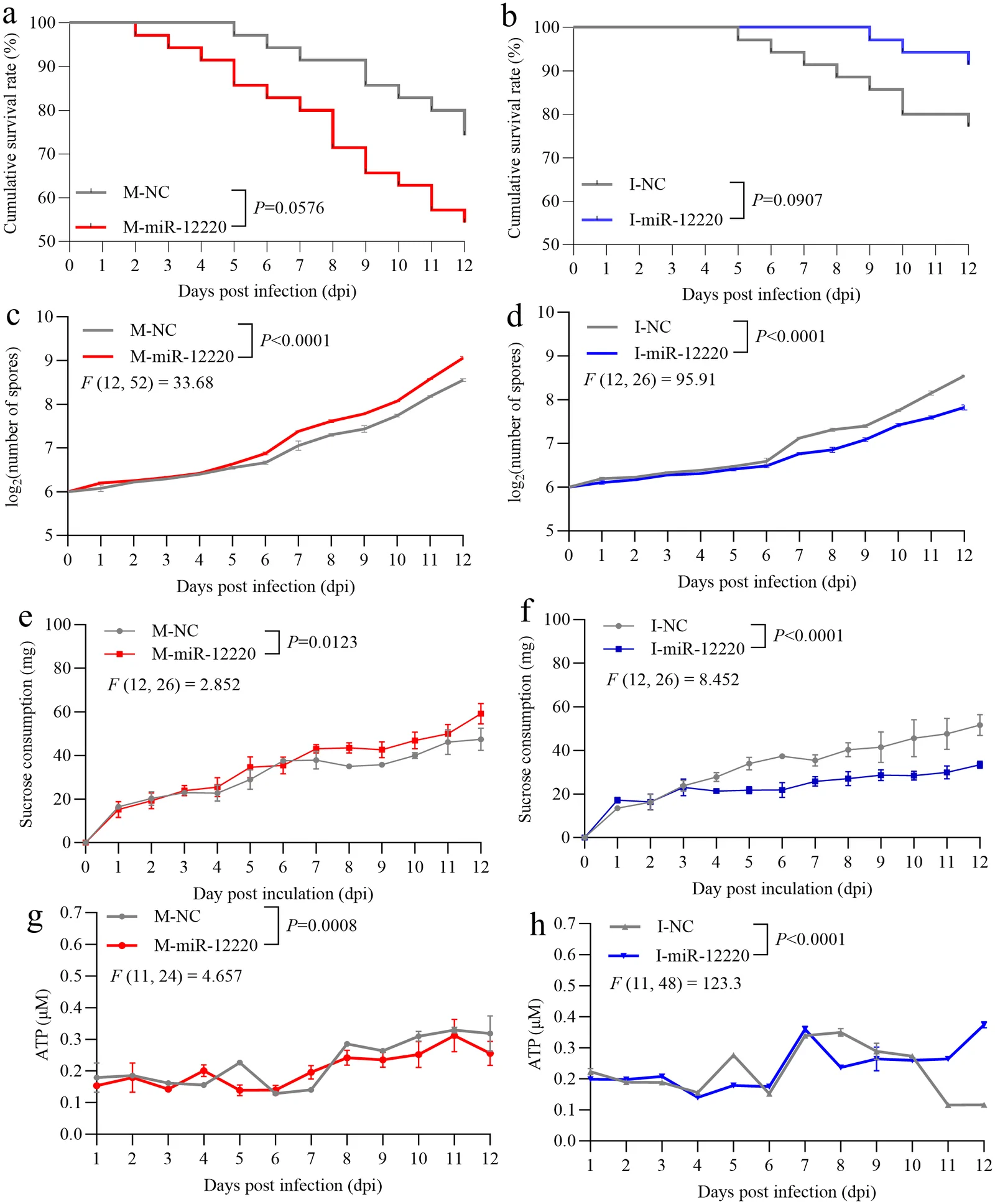
Effects of nce-miR-12220 overexpression and knockdown on survival, *N. ceranae* spore load, sucrose consumption, and midgut ATP content in infected *A. mellifera* workers. **(a-b)** Kaplan–Meier survival curve of infected workers following nce-miR-12220 overexpression and knockdown. **(c-d)** *N. ceranae* spore load from 1 to 12 dpi following nce-miR-12220 overexpression and knockdown. **(e-f)** Consumption of 50% (w/v) sucrose solution, expressed as milligrams per worker per day, by infected workers following nce-miR-12220 overexpression and knockdown. **(g-h)** Midgut ATP content following nce-miR-12220 overexpression and knockdown. Survival distributions were compared using the log-rank (Mantel–Cox) test. For panels **(c–h)**, statistical significance was assessed using a mixed-effects model for repeated-measures data. Data are presented as the mean ± SD of three biological replicates.

To further assess the biological effects of nce-miR-12220 during *N. ceranae* infection, worker survival, *N. ceranae* spore load, sucrose consumption, and midgut ATP content were evaluated following nce-miR-12220 overexpression or knockdown. Compared with the M-NC group, nce-miR-12220 overexpression was associated with a lower survival probability, although the difference did not reach statistical significance (*P* = 0.0576; Fig 6a). Conversely, workers in the I-miR-12220 group tended to show improved survival relative to the I-NC group, but the difference was also not significant (*P* = 0.0907; Fig 6b).

nce-miR-12220 overexpression significantly increased *N. ceranae* spore load compared with the M-NC group (*P* < 0.0001; Fig 6c), whereas nce-miR-12220 knockdown significantly reduced spore load relative to the I-NC group (*P* < 0.0001; Fig 6d). These findings support a role for nce-miR-12220 in promoting *N. ceranae* proliferation during infection.

Sucrose consumption also changed following nce-miR-12220 manipulation. Workers treated with mimic-miR-12220 consumed significantly more sucrose than those in the M-NC group across the examined period (*P* = 0.0123; Fig 6e). In contrast, nce-miR-12220 knockdown significantly reduced sucrose consumption compared with theI-NC group (*P* < 0.0001; Fig 6f).

Midgut ATP content was then measured to assess host energy status. nce-miR-12220 overexpression significantly decreased ATP content relative to the M-NC group (*P* = 0.0008; Fig 6g), whereas nce-miR-12220 knockdown significantly increased ATP content compared with the I-NC group (*P* < 0.0001; Fig 6h). Together, these results suggest that nce-miR-12220 promotes *N. ceranae* proliferation and is associated with altered feeding behavior and reduced host midgut energy reserves during infection.

### Discussion

Currently, study on miRNAs in microsporidian, especially *Nosema* species, is very limited. Following deep sequencing and bioinformatics, Huang’s group previously identified 9 miRNAs from *N. ceranae* [18]. A total of 10 miRNAs, including nce-miR-12220, were recently discovered in the *N. ceranae* spores by our team [23]. However, no intersection was observed in the documented miRNAs between Huang’s study and our work. Here, following expression pattern detection, target prediction and functional annotation of nce-miR-12220 [28], we for the first time conducted deep investigation of the nce-miR-12220-mediated regulatory mechanism underlying the *N. ceranae* infection of *A. mellifera* worker, providing a solid experimental evidence for the bee microsporidian infection regulated by miRNA. Microsporidia within the genus *Nosema* are characterized by their ability to produce spores with a distinct layered wall structure [29]. Notably, no mitochondrial structures were detected in the *N. ceranae* spores, the absence of mitochondria implied that *N. ceranae* cannot generate ATP via traditional methods such as oxidative phosphorylation or the tricarboxylic acid (TCA) cycle. It has been reported that microsporidia can directly import host ATP through ATP/ADP transport proteins and secure their energy supply by manipulating host metabolism like activation of the glycolysis, TCA cycle, and key enzyme expression [30]. Such a strategy exemplifies the adaptive evolution of parasitic organisms under extreme genomic reduction [31]. However, the details of the interaction between microsporidia and host mitochondria are not well understood. The mechanism by which *N. ceranae* hijacks the host energy metabolism presents an intriguing scientific question.

*N. ceranae* spores exist as dormant entities in the environment, invading hosts through polar filament ejection to infect midgut epithelial cells and injecting sporoplasm for proliferation [32]. nce-miR-12220 was significantly upregulated at 2 dpi (224.58%) followed by marked downregulation from 3 to 12 dpi [24]. This expression pattern suggests that nce-miR-12220 may play a role not only in the initiation of spore polar filament ejection and early infection but also in the modulation of fungal proliferation. MiRNAs typically exert biological effects on the host by negatively regulating target genes [33,34]. Recent evidence has suggested that some miRNAs could also positively regulate target gene expression and some crucial processes [35]. In a previous study, we found that nce-miR-12220 formed a regulatory network with 15 target genes, encompassing various physiological processes, such as energy metabolism and cellular structure [24]. Both *ATP-A* and *γ-tubulin* are critical genes associated with energy metabolism. *ATP-A*, as a component of vacuolar ATP synthase, is involved in energy transport across membranes and regulates numerous physiological processes in host cells [36,37]. *γ-tubulin* is a pivotal protein in microtubule organizing centers, playing essential roles in microtubule formation and cell cycle regulation [38]. Here, we detected that during the infection process of *N. ceranae*, the *ATP-A* expression was significantly upregulated at 2 dpi, followed by downregulation from 3 to 12 dpi (Fig 1a), consistent with the expression pattern of nce-miR-12220. Meanwhile, *γ-tubulin* was continuously downregulated from 2 to 12 dpi, demonstrating a similar trend to that of nce-miR-12220 and *ATP-A* [24]. These demonstrated that nce-miR-12220 was likely to positively regulate both *ATP-A* and *γ-tubulin*. In addition, the binding relationship between nce-miR-12220 and *ATP-A* (*γ-tubulin*) was verified by dual-luciferase reporter assays (Fig 1), offering strong evidence for the hypothesis that nce-miR-12220 participated in the *N. ceranae* infection process through regulating these target genes. In this work, it’s observed that the nce-miR-12220 overexpression significantly upregulated the expression of *ATP-A* and *γ-tubulin* (Fig 4c and 4e). Conversely, the knockdown of nce-miR-12220 significantly reduced the expression of both genes (Fig 4d and 4f). Together, these results further validated the regulatory role of nce-miR-12220 in the *N. ceranae* infection.

RBL, serving as a virulence factor of microsporidia, plays a essential part in the fungal infection [39]. Studies have shown that the RBL domain, acting as an adhesion protein, is an indispensable component in microsporidia’s infection process and a pivotal mediator in their binding to host cell surfaces [40,41]. This process facilitates the initial invasion of the microsporidia, representing a key step in the establishment of infection [41]. Our study demonstrated that silencing *ATP-A* or *γ-tubulin* led to a significant reduction in the expression level of *NcRBL* during 1–6 dpi (Fig 2), indicating that targeting these genes could interfere with microsporidian invasion by suppressing the *NcRBL* expression. Further analysis revealed that interference with *ATP-A* or *γ-tubulin* significantly increased survival rates (Fig 3b). In summary, these results demonstrated that silencing *ATP-A* or *γ-tubulin* could suppress the microsporidian proliferation and subsequently improve host survival, indicative of their vital functions in the *N. ceranae* infection.

The Toll signaling pathway plays a critical role in insect immune responses [42], serving as a key immune barrier particularly for insects against infections by pathogenic microorganisms (including bacteria, viruses, and fungi) [42]. Serpin-1a and serpin-6 regulate the Toll pathway immune homeostasis by synergistically inhibiting the Spätzle-processing enzyme CLIP2 in silkworm, *Bombyx mori*. For instance, infection with Rice stripe virus (RSV) can activate the Toll pathway in insect vectors, upregulating the expression of core pathway genes (e.g., *Toll*, *Dorsal*), thereby inhibiting viral proliferation [43,44]. In shrimp and *Drosophila*, the Toll pathway regulates the expression of immune effector molecules such as anti-lipopolysaccharide factors (ALFs) through the activation of transcription factor Dorsal [45]. In the present study, nce-miR-12220 overexpression coordinately decreased the transcript levels of *Cactus* and *dorsal*, whereas nce-miR-12220 knockdown produced the opposite expression pattern (Fig 5a-5d). The simultaneous downregulation of *Cactus* and *Dorsal* transcripts after nce-miR-12220 overexpression likely indicates broad transcriptional suppression or dysregulation of Toll-related immune signaling during infection, rather than canonical activation of the pathway.

Insect antimicrobial peptides (AMPs) are core components of the insect innate immune system, playing a pivotal role in resisting the invasion of pathogenic microorganisms and maintaining host health [46]. They are primarily produced by humoral immune responses; for example, after pathogenic infection, they are released from the fat body into the hemolymph, exerting direct killing effects on microorganisms such as bacteria, fungi, and viruses [47]. Defensin is an important class of cationic peptides in the insect antimicrobial peptide family, renowned for its broad-spectrum antimicrobial activity and regulatory role in innate immunity [48]. For instance, in honeybees (*Apis cerana cerana*), the expression of *Defensin* genes (e.g., *Defensin-1*) serves as a significant marker of immune responses, which undergoes significant changes under the influence of environmental factors such as insecticides (e.g., azadirachtin). Hymenoptaecin is a specific insect antimicrobial peptide, whose immune functions have been mainly studied in honeybees [49]. Similarly, the present study also found that overexpression of nce-miR-12220 could significantly lead to the downregulation of antimicrobial peptide genes such as *Hymenoptaecin* and *Defensin* (Fig 5).

The results indicated that nce-miR-12220 may promote the *N. ceranae* proliferation by controlling host immune response via regulation of these four critical immune genes. A similar strategy exists in the infection process of other microsporidia against their hosts. For instance, microsporidia utilize the nucleus-targeted effector EnP1, which translocates into the host nucleus via nuclear localization signals, to interact with host histone H2B. This interaction disrupts H2B monoubiquitination, thereby inhibiting the expression of p53 and impairing its regulation of downstream *SLC7A11*. Consequently, the ferroptosis resistance of host cells is enhanced, creating favorable conditions for the proliferation of microsporidia themselves [49].

*N. ceranae* lacks the classical mitochondrial structure and thus cannot generate ATP through mitochondria like other eukaryotes; instead, it must obtain energy from the host to sustain its own metabolic requirements [30]. Studies have reported a significant increase in sugar intake in bees infected with *N. ceranae* [50]. In the present study, nce-miR-12220 overexpression increased sucrose consumption and decreased midgut ATP content, while its knockdown produced the opposite effects (Fig 6e- 6h). These coordinated changes suggest that nce-miR-12220 is associated with altered host energy expenditure and ATP homeostasis during *N. ceranae* infection. Consistently, nce-miR-12220 overexpression increased *N. ceranae* spore load, whereas its knockdown reduced spore abundance (Fig 6c- 6d), supporting a role for this miRNA in promoting parasite proliferation. Although whole-midgut ATP measurements do not resolve the specific metabolic pathways involved, future parasite-specific ATP/ADP analyses and glycolytic enzyme assays may further clarify the underlying mechanism.

## Conclusion

**Fig 7:**
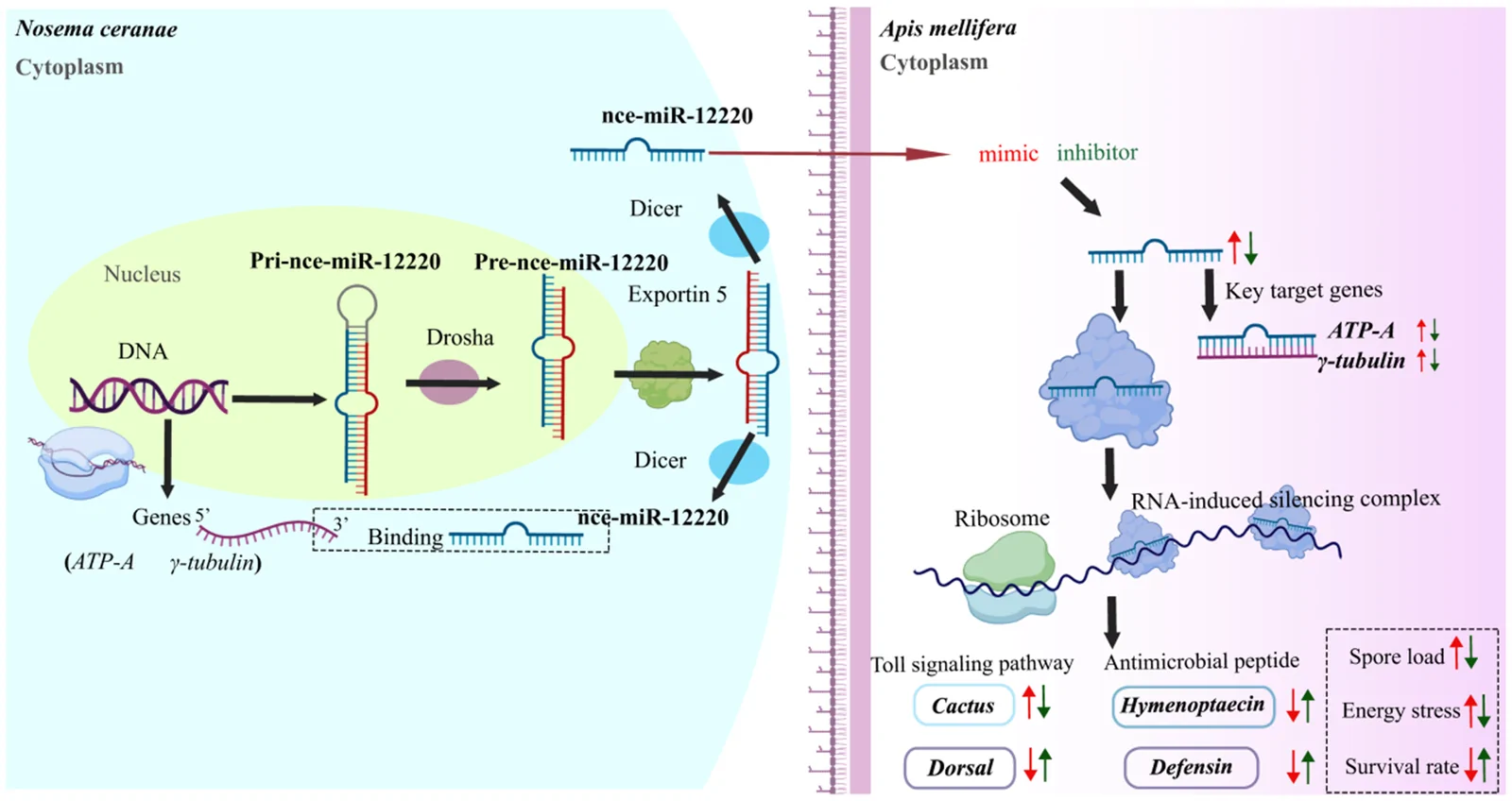
A working model of the *N. ceranae* infection of the *A. mellifera* worker regulated by nce-miR-12220.

In conclusion, nce-miR-12220 positively modulates the expression of *ATP-A* and *γ-tubulin* and enhances the microsporidian proliferation during the *N. ceranae* infection of *A. mellifera* workers, controlling host immune system through suppressing the Toll signaling pathway and antimicrobial peptide gene expression, and ultimately, influencing host energy expenditure as well as survival (Fig 7).

## Supporting information

S1 Table The primers and sequences used in this study. (DOCX)

S1 Raw Data Raw numerical data underlying the graphs presented in Figs 1–6. (ZIP)
