# Supplementary material for: nce-miR-12220 is a vital regulator for microsporidian to infect honeybee via positive modulation of ATP-A and γ-tubulin genes
Source: PLoS Pathog. 2026 Aug 17;22(8):e1014521. doi: 10.1371/journal.ppat.1014521 (PMC13492980; doi:10.1371/journal.ppat.1014521)
Supplement: Supplementary file 1 [file ppat.1014521.s001.docx]

Table S1 The primers and sequences used in this study

| Primer or sequence name | Sequence (5'–3') |
| --- | --- |
| nce-miR-12220-Loop | CTCAACTGGTGTCGTGGAGTCGGCAATTCAGTTGAGCCTAAAGA |
| nce-miR-12220-F | CGCGCGTTTAATTGTGAAACATG |
| nce-miR-12220-R | CTCAACTGGTGTCGTGGA |
| 5s rRNA-F | CGAGCGGTTTCCCATCTCAGTA |
| 5s rRNA-R | AAAACACCGGAACTCGTCAGCT |
| ATP-A-F | TCTGATGCTATTGTTTATGT |
| ATP-A-R | TCCTTCTACTGTTAGTTTTG |
| γ-tubulin-F | TTTCTCAACTATCTCCCG |
| γ-tubulin-R | ACATCCTTCTGCCTCTCT |
| Ncactin-F | ACAATGGTTCAGGTATCGTA |
| Ncactin-R | GTGCCTCATCTCCTACATAA |
| mimic-nce-miR-12220-sense | GACUCACGUCGACUGGGUGUCCG |
| mimic-nce-miR-12220-antisense | GACACECAGUCGACGUGAGUCUU |
| mimic-NC-sense | UUCUCCGAACGUGUCACGUTT |
| mimic-NC-antisense | ACGUGACACGUUCGGAGAATT |
| ATP-A-miR-12220-wt-F | CATAAAAAAACAGACTGAAGACATTTTTTACCAATTAATGAAAATGAAC |
| ATP-A-miR-12220-wt-R | TCGAGTTCATTTTCATTAATTGGTAAAAAATGTCTTCAGTCTGTTTTTTTATGAGCT |
| γ-tubulin-miR-12220-wt-F | CAAACAAAAAAGCAAGGCATCGCGGGTATCTGGATTATCCTTGTTGAAC |
| γ-tubulin-miR-12220-wt-R | TCGAGTTCAACAAGGATAATCCAGATACCCGCGATGCCTTGCTTTTTTGTTTGAGCT |
| ATP-A-miR-12220-mut-F | CATAAAAAAACAGGACTGGTGAGCCCCCCGAAGGCCGGCGAAAATGAAC |
| ATP-A-miR-12220-mut-R | CATAAAAAAACAGGACTGGTGAGCCCCCCGAAGGCCGGCGAAAATGAAC |
| γ-tubulin-miR-12220-mut-F | CAAACAAAAAAGAGGTTAGCATATTTCGCACTTGCCGCCCTTGTTGAAC |
| γ-tubulin-miR-12220-mut-R | TCGAGTTCAACAAGGGCGGCAAGTGCGAAATATGCTAACCTCTTTTTTGTTTGAGCT |
| siRNATP-A-A-sense | GCUGGAAGUAAACACACUUTT |
| siRNATP-A-A-antisense | AAGUGUGUUUACUUCCAGCTT |
| siRNA-γ-tubulin-sense | GCUUUGGAUCUAUGACUUUTT |
| siRNA-γ-tubulin-antisense | AAAGUCAUAGAUCCAAAGCTT |
| siRNA-scramble-sense | UUCUCCGAACGUGUCACGUTT |
| siRNA-scramble-antisense | ACGUGACACGUUCGGAGAATT |
| NcRBL-F | TGGTAGCGAGAGAAATAAGT |
| NcRBL-R | TCAAAGCAAGAACAAGAGTC |
| inhibitor-nce-miR-12220 | CGGACACECAGUCGACGUGAGUC |
| Inhibitor-NC | CAGUACUUUUGUGUAGUACAA |
| Cactus-F | CCTGGACTGTCTGGATGGTT |
| Cactus-R | TGGCAAACCCTTTCTCAATC |
| Dorsal-F | CTTTTATGTCGCGTGCAAAT |
| Dorsal-R | CGTTCTGTGTTCATTTCTCTTG |
| Hymenoptaecin-F | ACATAGAAATCGATCAGCTC |
| Hymenoptaecin-R | AACCAACACGATGAATTTCA |
| Defensin-F | GTCAGACACTTCTCAGTCTT |
| Defensin-R | TCTCAAAATGCTCAAGTGGCTC |
